# Smokers show increased fear responses towards safety signals during fear generalization, independent from acute smoking

**DOI:** 10.1038/s41598-022-12550-5

**Published:** 2022-05-24

**Authors:** Madeleine Mueller, Smilla Weisser, Jonas Rauh, Jan Haaker

**Affiliations:** 1 Department of Systems Neuroscience, University Medical Center Hamburg-Eppendorf (Germany), Martinistr.52, 20251 Hamburg, Germany; 2 Department of Psychiatry and Psychotherapy, Psychiatry Neuroimaging Branch, University Medical Center Hamburg-Eppendorf (Germany), Martinistr.52, 20251 Hamburg, Germany

**Keywords:** Risk factors, Fear conditioning

## Abstract

Smoking is highly prevalent among patients with anxiety disorders. Previous studies suggest that smokers show altered fear learning as compared to non-smokers. To test the effect of acute smoking on fear learning and generalization, we conducted a fear learning experiment online. 202 healthy subjects learned to differentiate a danger and a safe cue on day 1 and were tested for generalization of threat responses 24 h later. To see if the timing of smoking impacts fear learning, we formed three smoker groups with manipulations of acute smoking and withdrawal at different time-points (each group: n = 46) and one non-smoker control group (n = 64). Smoking manipulations contained a 6 h withdrawal after fear learning, smoking directly before or after fear learning. We found no group differences between smoker manipulation groups for fear learning or generalization. However, we found differences in fear generalization between smokers and non-smokers. Smokers showed increased fear ratings towards the stimulus that has been learned as safe and higher US expectancy to stimuli similar to the safe stimulus, when compared to non-smokers. Smoking might constitute a risk factor for impaired discrimination between danger and safety and smoking restrictions could be an effective way to reduce the risks of development or maintenance of anxiety disorders.

## Introduction

Anxiety disorders (AD) are among the most frequent mental disorders^1^. Patients suffering from AD, similar to other patients with psychiatric disorders, are more likely to smoke compared to healthy individuals (45.3% vs. 22.5% in healthy individuals)^2,3^. Furthermore, symptom severity in patients with posttraumatic stress disorder (PTSD) was positively correlated with the extent of nicotine dependence^4,5^. But not only patients with AD are affected by the influence of smoking on maladaptive responses to threats. Isensee et al. conducted a prospective longitudinal study that found a higher risk for the onsets of panic attacks in healthy individuals that smoked when compared to non-smoking individuals^6^.

While being a smoker seems to increase the risks of maladaptive aversive learning, it is an open question how acute smoking affects associative aversive learning mechanisms and might drive maladaptive responses to threats.

It is assumed that one central mechanisms for the development of AD is (maladaptive) aversive associative learning, when confronted with threats^7^. Associative learning of threat responses in the laboratory is commonly examined using classical fear conditioning protocols. When employing differential fear conditioning, a conditioned stimulus (CS+) is predictive for an aversive unconditioned stimulus (US), whereas another conditioned stimulus (CS−) is not. Subjects learn the differential prediction of the two CSs for the US and express conditioned threat responses to the CS+ in comparison to the CS−. The CS− is learned as a safety stimulus and it is therefore adaptive to inhibit conditioned threat responses to the CS−^8,9^.

Transfer of learned threat responses to novel objects can be very useful for coping with changing environments. Such transfer can be observed as generalization and therefore includes expression of threat responses to stimuli that resemble the CS+ and inhibition of responses to stimuli that are similar to the CS−. Such generalization can be examined across a gradient of stimuli between the CS+ and the CS−. A shallow generalization gradient indicates a stronger generalization between stimuli, whereas a steeper generalization gradient indicates a stronger discrimination between stimuli.

Disproportionate threat responses to stimuli that resemble the CS+ (overgeneralization^10^) have been reported in patients diagnosed with Generalized anxiety disorder (GAD)^11^.

A key question is if acute smoking affects associative aversive learning mechanisms by distorting the balance between expression of conditioned threat responses and their inhibition. Such effect has been found for acute nicotine (the active ingredient of cigarette smoke) on conditioned threat responses in mice. In particular, acute nicotine administration increased responses to conditioned cues and contexts, relative to saline injections^12^ and nicotine impaired the inhibition of conditioned threat responses, when situations are safe. Specifically, the discrimination between dangerous and safe contexts seems to be dose-dependently disrupted when acute nicotine was acutely administered before threat learning^13^.

There is further translational evidence that animal studies of chronic nicotine administration might resemble effects of smoking on threat learning in human individuals. Kutlu et al. found that mice show a reduced discrimination between CS+ and CS− during threat learning (nicotine in a chronic schedule), which was mirrored by lower CS discrimination in humans that were smokers^14^. Another study showed that smokers, as compared to non-smokers, had an impaired differentiation between danger and safety context when retrieving of conditioned threat memories^15^. While these studies underline that smokers show a deficit in safety learning, they cannot, however, delineate how acute smoking before or after learning might drive later deficits in retrieving safety information.

To this end, we employed an online experiment entailing a differential threat conditioning protocol and a generalization task (24 h later)^16,17^. With this study, we want to provide a deeper understanding of the effects of acute smoking, when comparing fear acquisition between smokers and non-smokers. Further, a generalization task should clarify if a disruption of safety learning transfers to novel stimuli. We expected that acute smoking before or after fear acquisition would lead to an impaired safety learning in fear acquisition and generalization, when compared to individuals restricted from smoking. Furthermore, we expected a deficit of safety learning in smokers, when compared to non-smokers.

## Results

### Fear acquisition

In order to establish that subjects learn to discriminate between CS+ and CS− during acquisition training, we examined US expectancy and fear ratings towards the CS+ , when compared to the CS−. We expected this pattern to be disrupted in regard to safety learning in groups that were acutely smoking before acquisition training and in general, when comparing smokers to non-smokers.

#### US expectancy results

##### Four groups

**Figure 1:**
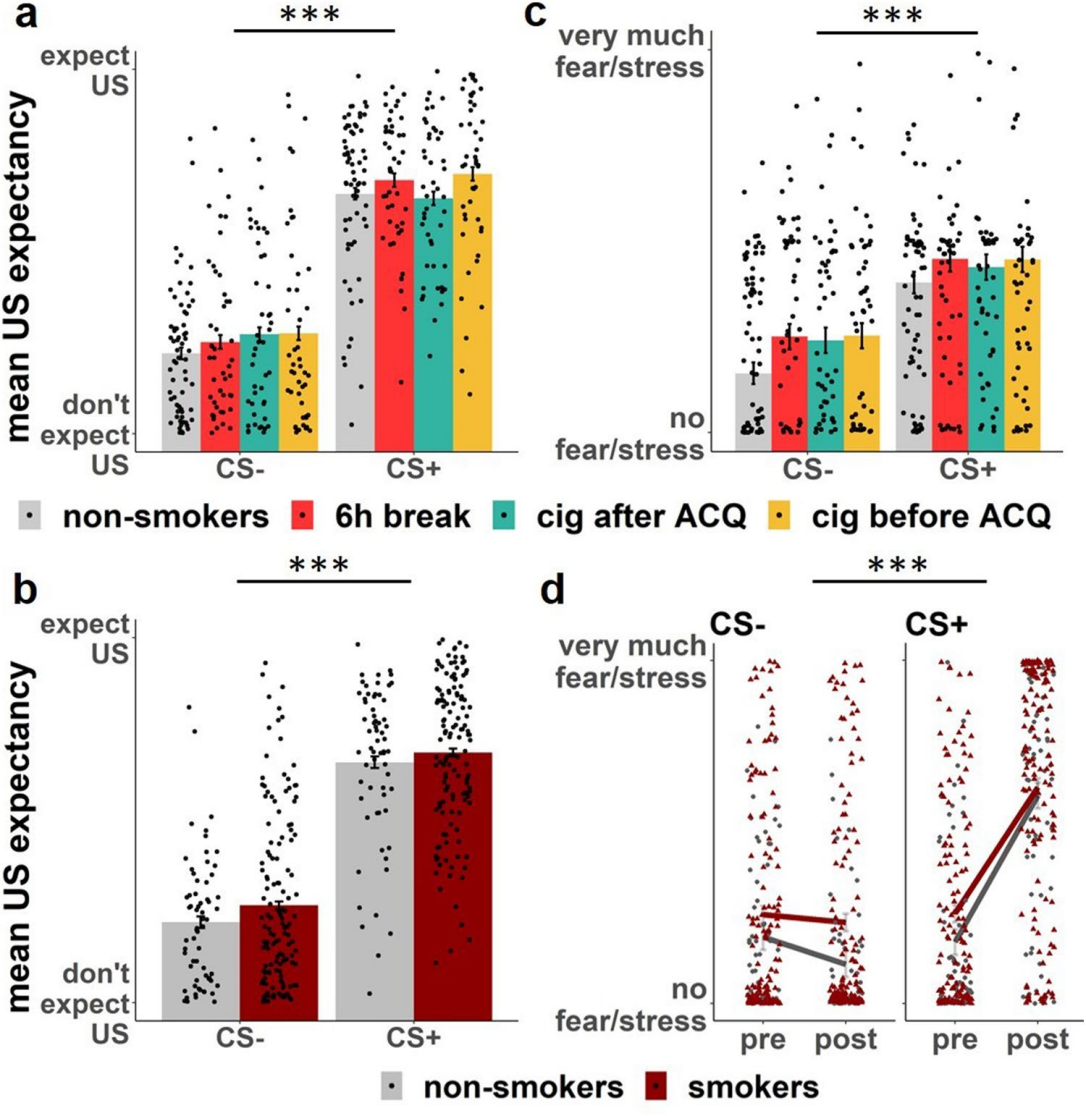
Rating Results Fear acquisition Day 1. Participants rated their US expectancy for every trial and their fear before and after fear acquisition. Individual representations indicate the mean for each subject. (**a**) Day 1 mean US expectancy of each group per stimulus. No group differences were found. (**b**) Day 1 mean US expectancy smokers versus non-smokers. No group differences were found. (**c**) Day 1 mean fear rating of each group. No group differences were found. (**d**) Day 1 fear rating smokers versus non-smokers. Following the colour, grey circles represent non-smokers and red triangles represent smokers. Both smokers and non-smokers showed an increased fear rating post acquisition towards the CS + , when compared to pre acquisition. Smokers showed generally increased fear ratings when compared to non-smokers. [***] indicates *p* < 0.001.

Participants learned to predict the US by the presence of the CS+ , indicated by a main effect of stimulus (F(1,4376.7) = 46.857, *p* < 0.001) with higher US expectancy for the CS+ as compared to the CS− (CS +–CS−: estimate = 3.8, SE = 0.078, z-ratio = 48.75, *p*_corr_ < 0.001) (Fig. 1a). Furthermore, we found a main effect of block (F(2,4380.9) = 12.894, *p* < 0.001) with an overall increasing US expectancy from block 1 to block 2 (block1–block2: estimate = − 0.5981, SE = 0.0956, z-ratio = − 6.256, *p*_corr_ < 0.001; block1–block3: estimate = − 0.6888, SE = 0.0959, z-ratio = − 7.184, *p*_corr_ < 0.001) and a stimulus by block interaction (F(2,4378.1) = 76.523, *p* < 0.001). The interaction consisted of higher differentiation between the CS+ and the CS− in block 2 when compared to block 1 (t(1989) = 5.86, *p*_corr_ < 0.001) as well as in block 3 when compared to block 2 (t(1990) = 4.543, *p*_corr_ < 0.001). Additionally, we found a block by group interaction (F(6,4381) = 2.394, *p* < 0.026), but follow-up post-hoc tests revealed no group differences.

##### Smokers versus non-smokers

When we compared smokers against non-smoking individuals, we found a trend towards a stimulus by block by group interaction (F(2,4388.6) = 2.75, *p* = 0.064), but follow-up post-hoc tests revealed no differences. We found a main effect of stimulus (F(1,4386.7) = 46.657, *p* < 0.001) with higher US expectancy for the CS+  as compared to the CS− (CS+–CS−: estimate = 3.85, SE = 0.083, z-ratio = 46.419, *p*_corr_ < 0.001; Fig. 1b). Furthermore, we found a main effect of block (F(2,4390.9) = 12.829, *p* < 0.001) with an increasing US expectancy from block 1 to block 2 (block1–block2: estimate = − 0.6365, SE = 0.102, z-ratio = − 6.271, *p*_corr_ < 0.001; block1–block3: estimate = − 0.7311, SE = 0.102, z-ratio = − 7.166, *p*_corr_ < 0.001) and a stimulus by block interaction (F(2,4388.2) = 76.194, *p* < 0.001). The interaction consisted of higher differentiation between the CS+ and the CS− in block 2 when compared to block 1 (t(1992) = 15.919, *p*_corr_ < 0.001) as well as in block 3 when compared to block 2 (t(1995) = 4.992, *p*_corr_ < 0.001).

#### Fear rating

##### Four groups

Participants rated higher fear for the CS+ as compared to the CS− (stimulus main effect (F(1,197) = 75.554, *p* < 0.001) and CS+ as compared to the CS− (t(804) = 9.005, *p*_corr_ < 0.001) (Fig. 1c). Furthermore, we found a main effect of time (F(1,197) = 89.335, *p* < 0.001) with an overall increase in fear ratings from pre ACQ to post ACQ (pre-post: t(804) = − 7.253, *p*_corr_ < 0.001) and a stimulus by time interaction (F(1,197) = 132.4, *p* < 0.001). The interaction consisted of higher differentiation between the CS+ and the CS− post ACQ when compared to pre ACQ (t(402) = 10.168, *p*_corr_ < 0.001). Additionally we found a main effect of group (F(3,197) = 2.701, *p* = 0.047), but follow-up post-hoc tests revealed no differences.

##### Smokers versus non-smokers

When comparing smokers to non-smokers, we found a main effect of group (F(1,199) = 7.754, *p* < 0.006) that indicated increased fear ratings in the smoker group, when compared to the non-smoker group (t(804) = 2.79, *p*_corr_ = 0.005; see Fig. S1). We found a stimulus main effect (F(1,199) = 74.254, *p* < 0.001) with higher fear ratings for the CS+ as compared to the CS− (t(804) = 9.005, *p*_corr_ < 0.001). Furthermore, we found a main effect of time (F(1,199) = 78.511, *p* < 0.001) with an increase in fear ratings from pre ACQ to post ACQ (pre–post: t(804) = − 7.253, *p*_corr_ < 0.001) and a stimulus by time interaction (F(1,199) = 134.803, *p* < 0.001) (Fig. 1d). The interaction consisted of higher differentiation between the CS+ and the CS− post ACQ when compared to pre ACQ (t(402) = 10.168, *p*_corr_ < 0.001).

We found that smokers show a generally increased fear rating during fear acquisition, when compared to non-smokers. Against our hypotheses, we did not find any group differences between smoking manipulation groups.

### Generalization test

To test for transfer of threat responses to the novel generalized stimuli, we employed a generalization in which we presented a gradient of new stimuli between the CS+ and the CS−. Now no stimulus was predictive for a US. We expected that retrieval of learned safety information (i.e., CS−) is altered in groups that were acutely smoking before and after acquisition training and in general, when compared to non-smokers.

#### US expectancy results

##### Four groups

**Figure 2:**
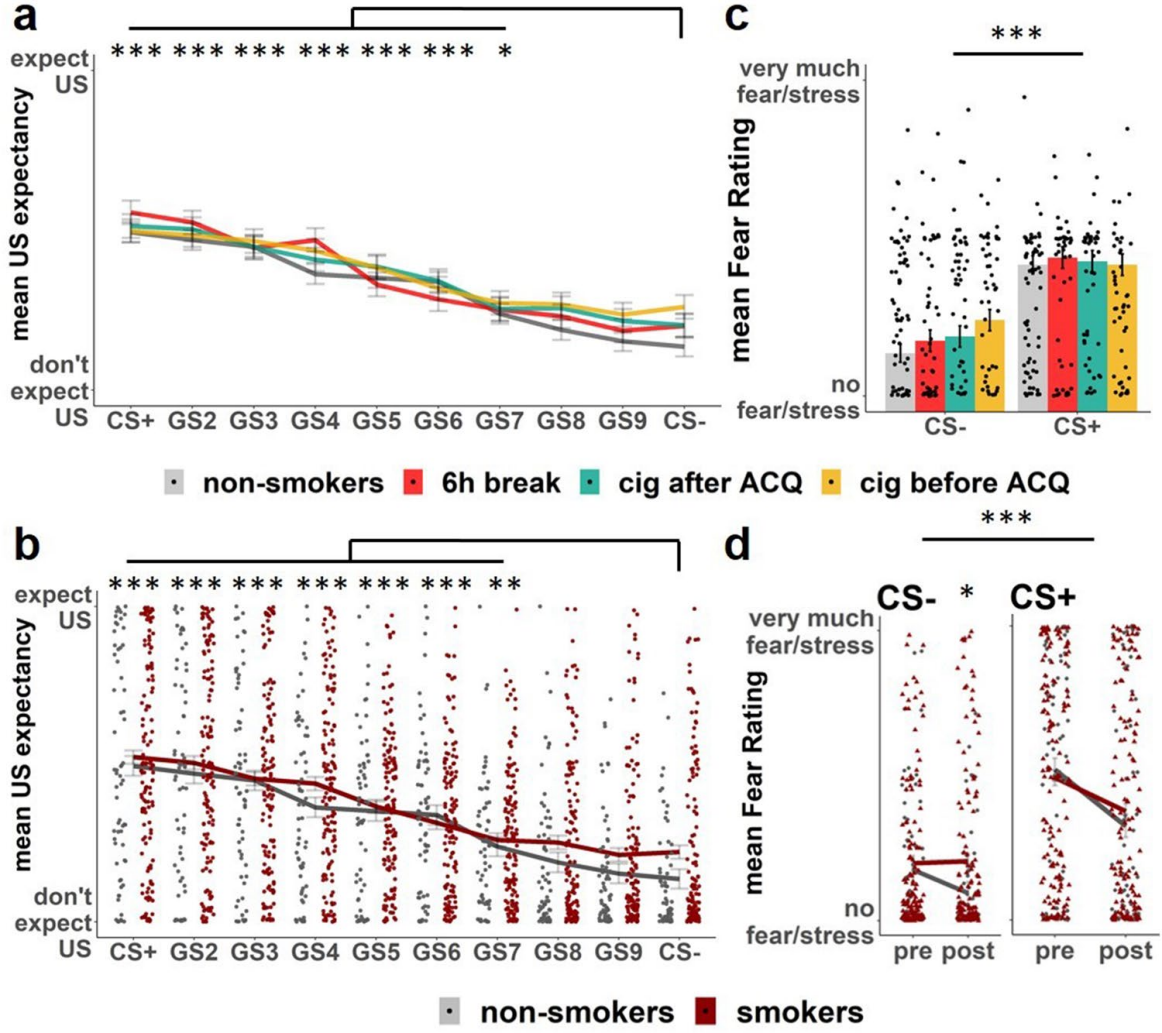
Rating results generalization test Day 2. Stimuli were presented in random order. Participants rated their US expectancy for every trial and their fear before and after the generalization test. Individual representations indicate the mean for each subject. (**a**) Day 2 mean US expectancy for each group per stimulus. Participants differentiated between the CS- and all stimuli between CS + and GS7 on Day 2. There were no group differences. (**b**) Day 2 mean US expectancy for all stimuli, smokers versus non-smokers. There were no group differences. (**c**) Day 2 mean fear rating for each group. No group differences were found. (**d**) Day 2 fear ratings smokers versus non-smokers. Following the colour, grey circles represent non-smokers and red triangles represent smokers. Non-smokers rated their fear/stress towards the CS- lower after the experiment when compared to smokers. [***] indicates *p* < 0.001. [**] indicates *p* < 0.01. [*] indicates *p* < 0.05.

Participants rated higher US expectancy for the CS+ as compared to each generalization-stimuli from GS3 to the CS− (stimulus main effect (F(9,3576.6) = 20.601, *p* < 0.001). Furthermore, participants rated lower US expectancy for the CS- as compared to each generalization-stimuli from GS7 to CS+ (estimates < − 2.92, for details see Table S1, Fig. 2a). Furthermore, we found a main effect of block (F(1,3576.6) = 4.959, *p* = 0.026) with a decrease of US expectancy from block 1 to block 2 (block1–block2: estimate = 0.927, SE = 0.0756, z-ratio = 12.264, *p*_corr_ < 0.001), which is likely an effect of no US presentation during this phase (resembling extinction training).

During the generalization test, we found that participants generalized their US expectancy to stimuli that resembled the CS+ and the CS-. Hence, we could identify two generalization groups of stimuli: Stimuli that were “CS+ like” (i.e., different from the CS− and not from the CS+ (CS+ , GS2, GS3)) and “CS− like” (i.e., different from the CS+ and not from the CS− (GS8, GS9, CS−)). Additionally, we identified a third group of stimuli that was different from both, the CS+ and the CS− and hence ambiguous stimuli (GS4–GS7). For grouping these stimuli we used solely non-smoker US expectancy ratings (for details see supplementary methods). Thereby, we defined how stimuli would be grouped in a “control” population. When using these generalization groups of stimuli in the mixed-model, we found a main effect of stimulus (F(2,3632.3) = 77.01, *p* < 0.001) with higher US expectancy to the CS + like in comparison to novel stimuli (estimate = 1.3, SE = 0.10, z-ratio = − 12.81, *p*_corr_ < 0.001) and higher US expectancy when comparing novel stimuli to CS− like stimuli (estimate = 1.36, SE = 0.09, z-ratio = 15.32, *p*_corr_ < 0.001). We found a main effect of block (F(1,3632.9) = 21.18, *p* < 0.001) with a decreasing US expectancy from block 1 to block 2 (estimate = 0.97, SE = 0.08, z-ratio = 11.76, *p*_corr_ < 0.001), as in the previous model. Additionally we found a stimulus group by block interaction (F(2,3632.1) = 3.4, *p* = 0.03) that was characterized by a decrease of US expectancy for each stimulus group decreasing from block 1 to block 2 (CS+ like: estimate = 1.44, SE = 0.171, z-ratio = 8.434, *p*_corr_ < 0.001; ambiguous: estimate = 0.99, SE = 0.11, z-ratio = 9.11, *p*_corr_ < 0.001; CS− like: estimate = 0.46, SE = 0.14, z-ratio = 3.32, *p*_corr_ = 0.002).

##### Smokers versus non-smokers

**Figure 3:**
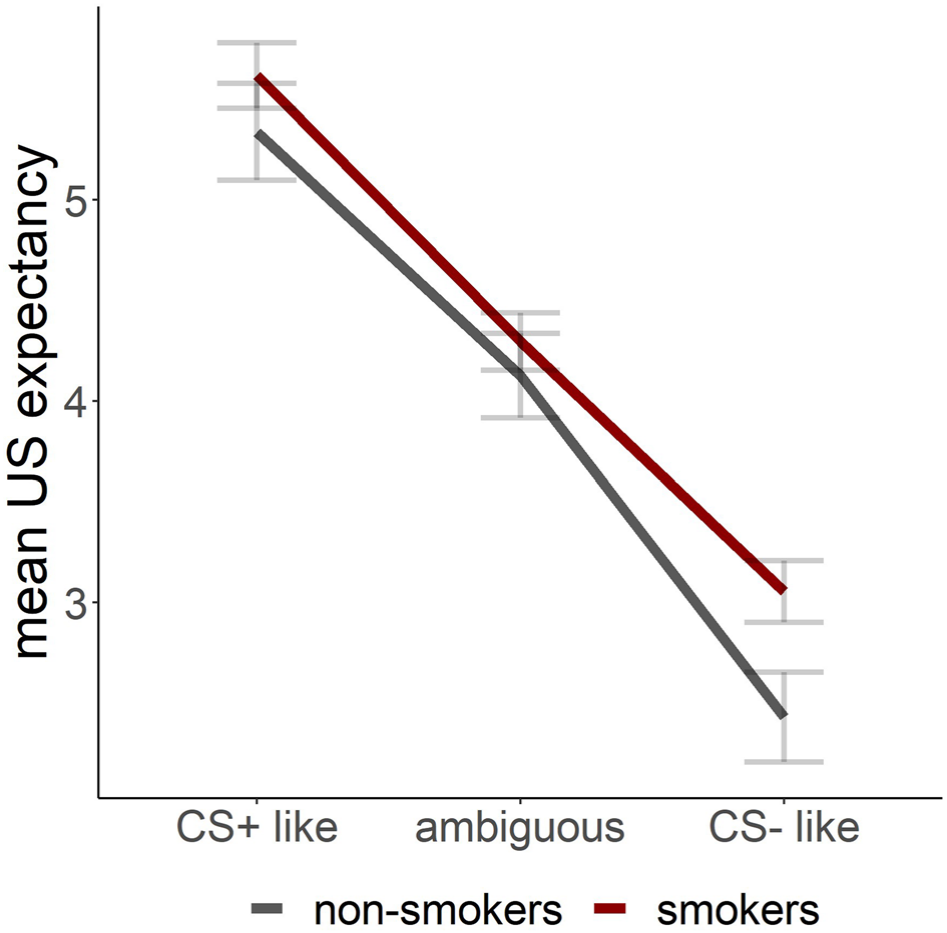
Day 2 mean US expectancy for grouped stimuli. Participants rated their US expectancy trialwise during the generalization test on day2 for CS+, CS− and the eight generalized stimuli in between (GS2–GS9). Rating results from the non-smoking group were used to define the grouping of stimuli. Stimuli that did not differ from CS+ were grouped as CS + like (CS+, GS2, GS3), stimuli that differed between CS+ and CS− were grouped as ambiguous (GS4–GS7) and stimuli that did not differ from CS− were grouped as CS-like (CS8, GS9, CS−). There is a trend towards a group difference between smokers and non-smokers when comparing CS− like stimuli during the generalization test on day 2. No differences were found between smokers and non-smokers for CS+ like and ambiguous stimuli.

We found a stimulus main effect (F(9,3614.6) 20.703, *p* < 0.001) with higher US expectancy for the CS+ as compared to each generalization stimulus that ranged from GS3 to CS− (estimates > 0.517) and lower US expectancy for the CS− as compared to each generalization stimulus that ranged from CS+ to GS7 (estimates < − 0.635, for details see Table S3, Fig. 2b). Furthermore, we found a main effect of block (F(1,3613.2) = 4.983, *p* = 0.026) with a decrease of US expectancy from block 1 to block 2 (block1–block2: estimate = 1.05, SE = 0.08, z-ratio = 13.135, *p*_corr_ < 0.001). When grouping the stimuli as CS+ like, CS− like and novel, we found an interaction for stimulus by group (F(2,3642.4) = 3.37, *p* = 0.035). The following post-hoc test revealed a trend towards an increased US expectancy rating in the smoker group when compared to the non-smokers for the CS− like stimulus group (Fig. 3; estimate = − 0.624, SE = 0.271, z-ratio = 2.307, *p*_corr_ = 0.063).

#### Fear rating

##### Four groups

We found a stimulus main effect (F(1,198) = 75.745, *p* < 0.001) with higher fear ratings for the CS+ as compared to the CS− (t(806) = 10.901, *p*_corr_ < 0.001) (Fig. 2c). Furthermore, we found a main effect of time (F(1,198) = 16.509, *p* < 0.001) with a decrease in fear ratings from pre generalization test to post generalization test (pre–post: t(806) = 3.35, *p*_corr_ < 0.001). Additionally, we found a stimulus by time interaction (F(1,198) = 11.791, *p* < 0.001). The interaction consisted of higher differentiation between the CS+ and the CS− pre generalization test when compared to post generalization test (t(402) = 2.6, *p*_corr_ = 0.01).

##### Smokers versus non-smokers

We found a stimulus main effect (F(1,200) = 75.013, *p* < 0.001) with higher fear ratings for the CS+ as compared to the CS− (t(806) = 10.901, *p*_corr_ < 0.001). Furthermore, we found a main effect of time (F(1,200) = 23.920, *p* < 0.001) with a decrease in fear ratings from pre generalization test to post generalization test (pre–post: t(806) = 3.35, *p*_corr_ < 0.001). We found a stimulus by time interaction (F(1,200) = 10.109, *p* = 0.002). The interaction consisted of higher differentiation between the CS+ and the CS− pre generalization test when compared to post generalization test (t(402) = 2.6, *p*_corr_ = 0.01) (Fig. 2d). Additionally, we found a time by group interaction (F(1,200) = 4.554, *p* = 0.034). An independent samples t-test revealed that the non-smoker group rated lower fear compared to the smoker group after the experiment towards the CS− (t(200) = − 2.857, *p*_corr_ = 0.02).

We found that smokers show an increased fear rating towards the safety cue (CS−) after fear generalization, when compared to non-smokers. Furthermore, we found a trend towards an increased US expectancy in CS- like stimuli in smokers, when compared to non-smokers. Again, we found no difference between groups that were acutely smoking before acquisition training or not.

## Discussion

Our results indicate a difference in fear learning and fear generalization between smokers and non-smokers that is not affected by acute smoking before or after acquisition training. Smokers show increased fear ratings towards the CS− after the generalization test when compared to non-smokers. Additionally, we found a trend to an increased US expectancy towards stimuli that were generalized as the CS− (CS− like stimuli) in smokers, when compared to non-smokers. Our results thereby suggest that safety learning processes in smokers might be impaired and that this impairment is even transferred to novel stimuli. Interestingly, we found these differences between smokers and non-smokers in a situation that required retrieval of safety information (i.e., no presentation of the US), but not during learning (i.e., fear acquisition). Our results are in line with findings from chronic nicotine administration in mice, which indicated increased freezing responses towards the CS−^14^. The experiments in rodents further suggest that nicotine administration throughout fear acquisition and extinction delayed safety learning and inhibition of conditioned threat responses during extinction training^18^. Additionally, the translation of this finding to humans revealed reduced CS-discrimination in smokers when compared to non-smokers, along with reduced CS-discrimination in mice^14^. Our finding that smokers show an impairment in retrieving safety information is further in line with a previous study that indicated impaired contextual inhibition in a safe context in smokers when compared to non-smokers^15^. Hence, our results pinpoint the influence of smoking to increased fear ratings and US expectancy ratings towards stimuli that have been learned as safe.

Against our hypothesis, we did not find an effect of any of our interventions on acute smoking (i.e., groups that smoked directly before or after the acquisition) and even withdrawal (i.e., control group that required a restriction of smoking for 6 h after acquisition) on fear learning or generalization. We furthermore could not find differences in aversive learning or the generalization test with respect to withdrawal symptoms or nicotine dependency between smokers (for details see supplement).

As a main effect, smokers showed generally increased fear ratings when compared to non-smokers, but we found no main group differences in the US expectancy or anxious temperament (STAI-trait anxiety). This indicates that there is no support for increased trait anxiety in smokers in our sample when compared to non-smokers (for details see supplement). Previous studies have found inconsistent results on whether smoking influences trait anxiety in humans (see for increased trait anxiety in smokers:^19^; see for no difference of trait anxiety between smokers and non-smokers:^20^).

In the generalization test on day 2, we could identify generalization of US expectancy to novel stimuli that were similar to the CS+ or CS− and hence analysed as CS+ like, CS− like or ambiguous stimuli. Interestingly smokers showed a trend towards an increased US expectancy to the CS− like stimuli, when compared to non-smokers. Both, US expectancy ratings and fear ratings decreased over time during the generalization test, which indicates extinction learning. This decrease in US expectancy was found for CS+ like and ambiguous stimuli in both smokers and non-smokers. However, the decrease of the CS− like stimuli was slightly weaker in smokers. In line with these findings for US expectancy are also the results of the fear ratings. Similarly to US expectancy, we found that smokers showed no decrease in rated fear during the generalization test to the CS−, whereas non-smokers did decrease their ratings. No differences in the decrease of fear ratings between groups were found for the CS+.

Our results suggest increased fear ratings and US expectancy in smokers when no US was present (generalization test) to a stimulus that was learned safe (CS−) when compared to non-smokers. This effect in smokers could be linked to maladaptive threat responses that are observed in individuals that suffer from pathological anxiety. Previous meta-analyses found that patients with anxiety-related disorders show an increased fear response during fear acquisition towards the CS− when compared to healthy controls^9,21^. Hence, our results in smokers resemble learning deficits in individuals with anxiety-related disorders. Thereby, our results might highlight a possible linkage between smoking and pathological anxiety: impairments in learning and retrieving safety information. Future studies are required to clarify if smoking causally leads to such learning deficits or the other way around.

One interesting aspect from our results might be relevant for the prevention and treatment of pathological anxiety: We would advocate to promote smoking cessation programs in order to reduce the risk of maladaptive threat responses in the long run. Our results support no effect of smoking restriction and withdrawal symptoms on threat responses. Hence, the process of quitting should not lead to exaggerated threat responses, per se. In fact, clinical studies that employed a smoking cessation treatment in parallel to mental health care for PTSD patients reported high rates of smoking abstinence and successful reduction of PTSD symptoms^22^. Hence, individuals that might be diagnosed with AD or exhibit other symptomatology that involves impaired safety learning might also benefit from smoking cessation programs.

Our results have several limitations. As this is an online study, there is an inherently reduced control of the participants with respect to their adherence to the study protocol (i.e. smoking manipulations). We informed the participants that there are no consequences if they for example could not fulfil the 6 h smoke break, but we encouraged them to honestly report if they managed to restrict smoking for 6 h, or not. Several participants reported that they have not followed instruction and were regrouped. This might seem like a big disadvantage of an online study, but it also provides a chance to investigate participants at home in their usual smoking environment. Most participants also followed the timeframe instructions of 24 h between day 1 and day 2 very precisely, which indicates a general adherence to instructions.

In sum, we found that smoking leads to increased fear responses towards the safety stimulus during and after the generalization test. No group differences were found during fear acquisition. Manipulations acute smoking and withdrawal at different time-points seem to make no difference in aversive learning, however the crucial point is if a person is a smoker or not. Smoking therefore might be considered a risk factor for impaired safety learning and ultimately enhances the individual development or maintenance of pathological anxiety. However, further investigations are needed to specify the causal effect of smoking and its active ingredient nicotine on fear learning in humans. Nevertheless, one clear recommendation for the reduction of the risk to develop maladaptive threat responses is to quit smoking.

## Material and methods

### Participants

For this online study 273 participants were recruited online. Healthy individuals between 18 and 65 years with no self-reported diagnoses of neuropsychiatric disorders, who consume less than 15 units alcohol per week and no illegal drugs could participate. The final sample for analysis consisted of 202 subjects (female: N = 125, mean age = 29.47 ± 9.81, Table 1). Participants were included into the final data set if the experiment on day 2 was started 24 h after starting day 1 (with a tolerance of 6 h before and after the start). Participants had to rate the US as more unpleasant than the nUS, which was defined by a mean rating of the 3 USs being at least 1 point higher than the mean rating of the 3 nUSs on a scale of 1 to 10. furthermore, participants with an incomplete data set were excluded (for an overview see Fig. S2). All participants gave written, informed consent and the experiment was approved by the local ethics committee (Ethikkommission der Ärztekammer Hamburg PV 5514). The subjects received 15€ as reimbursement for completing both days of the study. All research was performed in accordance with the relevant guidelines and regulations.

**Table 1:** Demographics for each group separately and for all smoker groups combined.

	Mean (SD) Non-smokers Group 1	Mean (SD) Smokers Group 2	Mean (SD) Smokers Group 3	Mean (SD) Smokers Group 4	Mean (SD) Smokers (Groups 2, 3 & 4)
Sample size	64	46	46	46	138
Age [years]	29.38 (10.47)	27.96 (8.49)	30.2 (10)	30.37 (9.59)	29.51 (9.44)
Gender	Females: 43 Males: 21	Females: 31 Males: 15	Females: 27 Males: 19	Females: 24 Males: 22	Females: 82 Males: 56
Coffee consumption [cups/day]	1.198 (1.07)	1.22 (1.21)	1.34 (1.05)	1.7 (1.358)	1.42 (1.22)
Alcohol consumption [drinks/week]	1.43 (1.98)	2.58 (2.72)	2.7 (2.69)	3.28 (3.24)	2.85 (2.91)
Fagerström [sum score]	–	1.96 (1.88)	3.3 (2.51)	2.61 (2.35)	2.62 (2.33)
STAI-T [sum score]	43.14 (5.68)	44.59 (9.39)	44.80 (6.05)	44.96 (6.79)	44.78 (7.55)

Group 1 contains only non-smokers, group 2 contains smokers that took a smoking break of 6 h after fear acquisition on day 1, group 3 contains smokers that smoked a cigarette directly after fear acquisition on day 1 and group 4 contains smokers that smoked a cigarette directly before fear acquisition on day 1. The last column contains all smokers from groups 2, 3 and 4 combined.

### Groups

**Figure 4:**
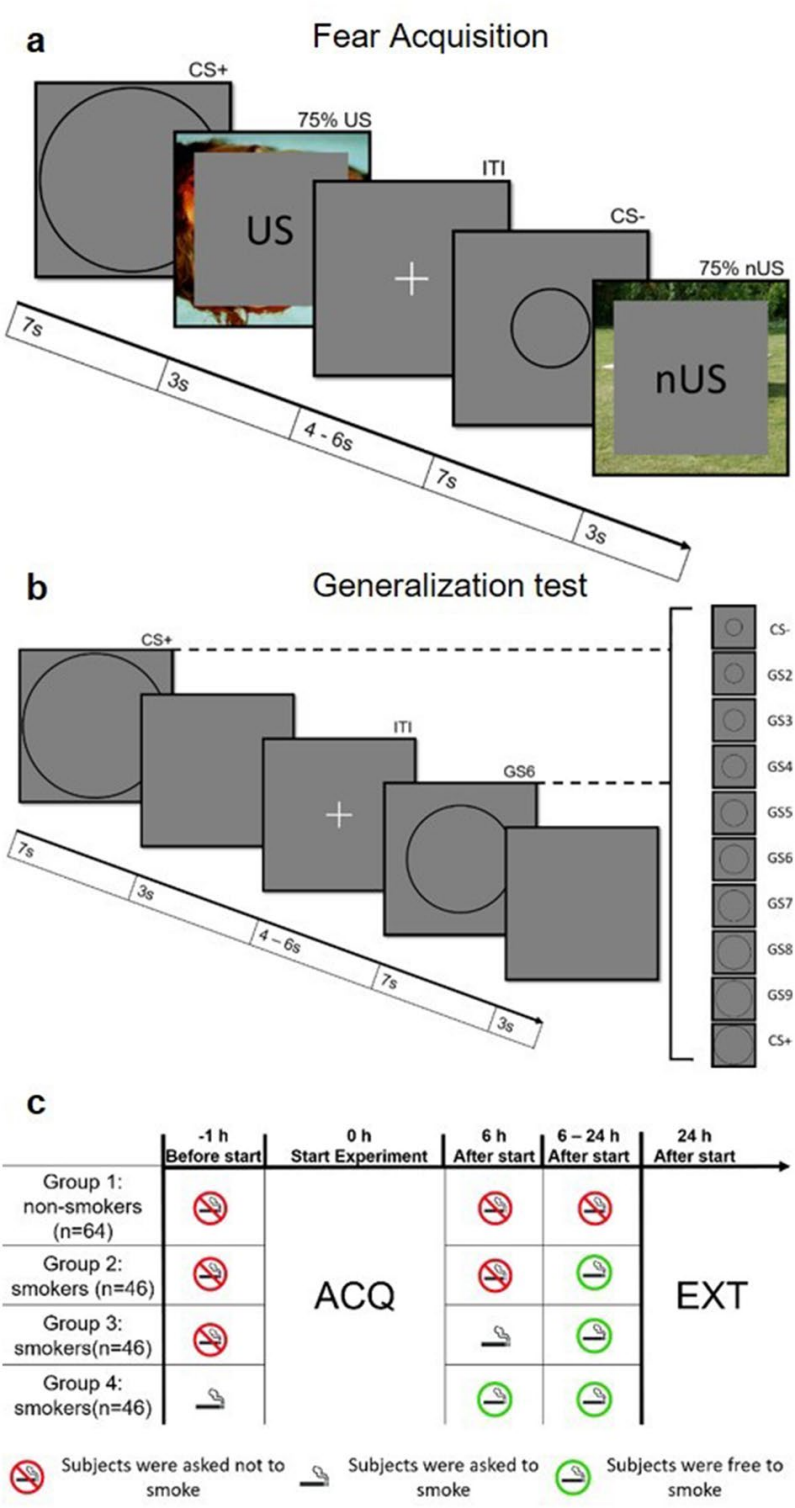
Experimental procedure. (**a**) Day 1: Fear acquisition. The CS + was followed in 75% of the time by an aversive picture, which served as US. The CS− was followed in 75% of the time by a neutral picture, which served as nUS. When not followed by a picture, CSs were fading into the grey background (1 s). Per trial a CS was presented for 7 s followed by a 1 s overlap of CS and US/nUS and then a 2 s only presentation of US/nUS. After each trial the ITI was presented (jittered between 4 and 6 s). Size of rings serving as CS + and CS− was counterbalanced. (**b**) Day 2: Generalization test. In addition to CS + and CS- serving as extremes, 8 rings of gradually increasing size were introduced as generalized stimuli (GS). After a 7 s presentation the CS or GS faded into the grey background. After each trial the ITI was presented (jittered between 4 and 6 s). (**c**) Group formation. The non-smoking group (group 1) did not smoke at all during the experiment. Group 2 (smokers) was asked not to smoke 1 h before and 6 h after the experiment on day 1. Group 3 (smokers) was asked not to smoke 1 h before the experiment and to smoke directly after the experiment on day 1. Group (smokers) 4 was asked to smoke directly before the experiment on day 1.

Four experimental groups were included in this study (Fig. 4c). The first group consisted of only non-smoking subjects (N = 64). The second group consisted of smoking subjects, who were instructed to have a 6-h smoking break after completing the acquisition (ACQ) on day 1 (N = 46). The third group consisted of smoking subjects, who were instructed to smoke after completing the acquisition on day 1 (N = 46). The fourth group consisted of smoking subjects, who were instructed to smoke a cigarette directly before starting the acquisition (N = 46). Subjects who were originally allocated to group 2, but did not take a 6 h smoking break were regrouped into group 3 for analyses of the final data set. Smokers were pseudo-randomly assigned to one of the smoker manipulation groups.

### Stimuli material

Two black rings with a smaller and a larger diameter (CS1: 5 cm; CS2: 11.75 cm) served as conditioned Stimuli (CS). CS1 and CS2 were counterbalanced as CS+ or CS−. For the Generalization test, additional eight generalized stimuli (GS2-–GS9) with an increasing 15% ring size between CS1 and CS2 were presented^16^. A black fixation cross served as the ITI.

Three pictures from the International affective picture system (IAPS) database^23^ that were rated as unpleasant (^24^, #3001, #3030, #3051) were chosen as unconditioned Stimuli (US) that followed upon the CS+ . Three different pictures that were rated neutral (#7009, #7026, #7175) were chosen as neutral US (nUS) that followed upon the CS−.

### Procedure

The study took place on 2 consecutive days with a temporal difference of 24 h (± 6 h). On the first day participants filled out a questionnaire containing a Fagerström test for nicotine dependence (FTND) and the State-Trait Anxiety Inventory (STAI-S/STAI-T) and gave information on age, sex, alcohol and coffee consumption and smoking habit. Questionnaires were completed on www.soscisurvey.de^25^. Directly after completing the questionnaires, participants started the behavioural experiment.

The experiment on day 1 contained a habituation phase in which the CS+ and CS− were presented once without being followed by the US/nUS. Day 1 further entailed an acquisition phase with three blocks, each consisting of four CS+ and four CS− presentations. Blocks were presented randomized. The CS+ presentation was followed by a US picture in nine out of twelve trials (75% reinforcement rate). Similarly, a nUS picture followed in 75% of the CS− trials. In 25% of the trials, the CSs faded out without US/nUS presentation. Each trial started with a 7 s CS presentation that was followed by a 3 s US/nUS presentation and ended with an ITI presentation (jittered between four to six seconds) (Fig. 4a).

Behavioural experiments were completed with PsychoPy3^26^. On the second day participants started again with a questionnaire containing STAI-S and a report on their overnight sleep. If the participant was sorted into the second group, they additionally answered if they managed to take a smoking break of 6 h after the acquisition and if not, when they started smoking again. Those participants were also asked if they noticed any withdrawal symptoms between day 1 and day 2.

Similar to the day before, participants started the behavioural experiment after finishing the questionnaires. The experiment on day 2 contained the generalization test with two blocks including one CS+ and one CS− presentation, as well as one GS2 to GS9 each. That concludes to ten trials per block, which were presented in random order. No CS or GS was followed by a US/nUS picture (Fig. 4b). The experiment ended with a CS/US identification where participants decided which CS or GS was paired with the US on day 1 (or if none of the stimuli were paired with the US). Participants had the possibility to stop the experiment at any time.

### Ratings

Participants rated their fear when confronted with CS+ and CS− on a continuous scale between 1 (no fear/stress) and 10 (very much fear/stress) before and after the acquisition phase on day 1, as well as before and after the generalization test on day 2.

On both days, participants rated their US expectancy on each trial (two to six seconds after CS onset) on a continuous scale from 1 (I don’t expect an unpleasant picture) to 10 (I definitely expect an unpleasant picture).

After generalization test and fear rating participants were asked about how unpleasant they perceived the US/nUS photos on a continuous scale from 1 (not unpleasant) to 10 (very unpleasant) (for details see Fig. S3). Additionally, participants were asked for their avoidance (how often they looked away from the screen) and their awareness (if they noticed a connection between the CS and the US/nUS on day 1).

### Data analysis

For analysis of the US expectancy we calculated linear mixed effect models in R using lme4 package^27^. The dependent variable in our model were the US expectancy ratings. Random intercepts and slope for subjects were entered in our model. The fixed effects in our model was the interaction between CS-types (CS+ and CS−), blocks (one to three) and groups (one to four; for smoker vs. non-smoker: one to two) (lmer (RatingResults ~ (1|participants) + stimuli*block*group)).Then an F-test with a Kenward-Roger approximation for degrees of freedom was performed in the form of an ANOVA type 3 calculation^28^. To further test the results estimated marginal means (EMMs) were computed using the emmeans package as post-hoc tests and p-values were corrected for multiple comparisons using Bonferroni-Holms method. Results with z-ratio are asymptotic results.

Fear ratings were analysed in Jasp using a repeated-measures ANOVA type 3 with repeated measures factors CS-type and time-point, as well as the between subject factor group. Follow-up tests were calculated by independent sample t-tests and were Bonferroni-Holm corrected.

## Supplementary Information

Supplementary Information.

## Data Availability

Data generated from this study are available from the authors upon reasonable request.

## References

[1] Bandelow B, Michaelis S (2015). Epidemiology of anxiety disorders in the 21st century. Dialogues Clin. Neurosci..

[2] Kutlu MG, Gould TJ (2015). Nicotine modulation of fear memories and anxiety: Implications for learning and anxiety disorders. Biochem. Pharmacol..

[3] Ziedonis D (2008). Tobacco use and cessation in psychiatric disorders: National Institute of Mental Health report. Nicotine Tob. Res..

[4] Baschnagel JS, Coffey SF, Schumacher JA, Drobes DJ, Saladin ME (2008). Relationship between PTSD symptomatology and nicotine dependence severity in crime victims. Addict. Behav..

[5] Thorndike FP, Wernicke R, Pearlman MY, Haaga DAF (2006). Nicotine dependence, PTSD symptoms, and depression proneness among male and female smokers. Addict. Behav..

[6] Isensee B, Wittchen H-U, Stein MB, Höfler M, Lieb R (2003). Smoking increases the risk of panic: Findings from a prospective community study. Arch. Gen. Psychiatry.

[7] Pittig A, Treanor M, LeBeau RT, Craske MG (2018). The role of associative fear and avoidance learning in anxiety disorders: Gaps and directions for future research. Neurosci. Biobehav. Rev..

[8] Lonsdorf TB (2017). Don’t fear ‘fear conditioning’: Methodological considerations for the design and analysis of studies on human fear acquisition, extinction, and return of fear. Neurosci. Biobehav. Rev..

[9] Lissek S (2005). Classical fear conditioning in the anxiety disorders: A meta-analysis. Behav. Res. Ther..

[10] Dunsmoor JE, Paz R (2015). Fear generalization and anxiety: Behavioral and neural mechanisms. Biol. Psychiat..

[11] Lissek S (2014). Generalized anxiety disorder is associated with overgeneralization of classically conditioned fear. Biol. Psychiat..

[12] Davis JA, Gould TJ (2007). β2 subunit-containing nicotinic receptors mediate the enhancing effect of nicotine on trace cued fear conditioning in C57BL/6 mice. Psychopharmacology.

[13] Kutlu MG, Oliver C, Gould TJ (2014). The effects of acute nicotine on contextual safety discrimination. J. Psychopharmacol..

[14] Kutlu MG (2018). Nicotine exposure leads to deficits in differential cued fear conditioning in mice and humans: A potential role of the anterior cingulate cortex. Neurosci. Lett..

[15] Haaker J (2017). Where there is smoke there is fear—impaired contextual inhibition of conditioned fear in smokers. Neuropsychopharmacology.

[16] Struyf D, Zaman J, Hermans D, Vervliet B (2017). Gradients of fear: How perception influences fear generalization. Behav. Res. Ther..

[17] Lissek S (2008). Generalization of conditioned fear-potentiated startle in humans: Experimental validation and clinical relevance. Behav. Res. Ther..

[18] Elias GA, Gulick D, Wilkinson DS, Gould TJ (2010). Nicotine and extinction of fear conditioning. Neuroscience.

[19] Pietras T, Witusik A, Panek M, Szemraj J, Górski P (2011). Anxiety, depression and methods of stress coping in patients with nicotine dependence syndrome. Med Sci Monit.

[20] Henry SL, Jamner LD, Whalen CK (2012). I (should) need a cigarette: Adolescent social anxiety and cigarette smoking. Ann. Behav. Med..

[21] Duits P (2015). Updated meta-analysis of classical fear conditioning in the anxiety disorders. Depress. Anxiety.

[22] McFall M (2010). Integrating tobacco cessation into mental health care for posttraumatic stress disorder<subtitle>a randomized controlled trial</subtitle>. JAMA.

[23] Lang, P. J., Bradley, M. M., & Cuthbert, B. N. *International affective picture system (IAPS): Technical manual and affective ratings*. NIMH Center for the Study of Emotion and Attention, 1(39–58) (1997).

[24] Xu Z (2017). Selecting pure-emotion materials from the International Affective Picture System (IAPS) by Chinese university students: A study based on intensity-ratings only. Heliyon.

[25] Leiner, D. J. SoSci Survey (Version 3.1.06) [Computer software]. Available at https://www.soscisurvey.de (2019).

[26] Peirce JW (2007). PsychoPy—psychophysics software in Python. J. Neurosci. Methods.

[27] Bates, D., Mächler, M., Bolker, B., & Walker, S. Fitting linear mixed-effects models using lme4. arXiv preprint arXiv:1406.5823 (2014).

[28] Figner, B. *et al.* Standard Operating Procedures For Using Mixed-Effects Models. A Principled Workflow from the Decision. Available at http://decision-lab.org/wp-content/uploads/2020/07/SOP_Mixed_Models_D2P2_v1_0_0.pdf(2020).

