# Supplementary material for: Smokers show increased fear responses towards safety signals during fear generalization, independent from acute smoking
Source: Sci Rep. 2022 May 24;12:8692. doi: 10.1038/s41598-022-12550-5 (PMC9130119; doi:10.1038/s41598-022-12550-5)
Supplement: Supplementary file 1 — Supplementary Information. [file 41598_2022_12550_MOESM1_ESM.pdf]

Smokers show increased fear responses towards safety signals during fear generalization, independent from acute smoking

Supplementary Material

Madeleine Mueller[1*], Smilla Weisser[1], Jonas Rauh[1,2] & Jan Haaker[1]

[1] University Medical Center Hamburg-Eppendorf (Germany), Department of Systems Neuroscience

[2] University Medical Center Hamburg-Eppendorf (Germany), Department of Psychiatry and Psychotherapy, Psychiatry Neuroimaging Branch

*Corresponding author: Madeleine Mueller; Martinistr.52, 20251 Hamburg, Germany; mad.mueller@uke.de; phone: +49 (0) 40 7410 – 57867; Fax: +49 (0) 40 7410-59955

Number of pages: 7, number of figures/tables: 7

Supplementary Results

Table S1: Day 2 US expectancy post-hoc tests between stimuli for all groups. Results were corrected for multiple comparisons using Bonferroni-Holms method.

Stimuli	Estimate	SE	z-ratio	p_{corr}
**CS+ - GS3**	0.517	0.179	3.36	0.01
**CS+ - GS4**	0.969	0.178	5.143	<0.001
**CS+ - GS5**	1.35	0.18	8.083	<0.001
**CS+ - GS6**	1.637	0.178	10.417	<0.001
**CS+ - GS7**	2.333	0.179	13.959	<0.001
**CS+ - GS8**	2.596	0.18	14.864	<0.001
**CS+ - GS9**	2.93	0.178	16.995	<0.001
**CS+ - CS-**	2.969	0.178	16.927	<0.001
**CS- - GS2**	-2.777	0.178	-15.86	<0.001
**CS- - GS3**	-2.452	0.179	-13.46	<0.001
**CS- - GS4**	-1.999	0.177	-11.804	<0.001
**CS- - GS5**	-1.619	0.179	-8.643	<0.001
**CS- - GS6**	-1.331	0.178	-6.456	<0.001
**CS- - GS7**	-0.635	0.178	-2.92	0.032

Table S2: Day 2 US expectancy post-hoc tests between stimuli for smokers vs. non-smokers. Results were corrected for multiple comparisons using Bonferroni-Holms method.

stimuli	Estimate	SE	z-ratio	p_{corr}
**CS+ - GS3**	0.517	0.179	2.881	0.04
**CS+ - GS4**	0.969	0.178	5.451	<0.001
**CS+ - GS5**	1.35	0.18	7.506	<0.001
**CS+ - GS6**	1.637	0.178	9.184	<0.001
**CS+ - GS7**	2.333	0.179	13.06	<0.001
**CS+ - GS8**	2.596	0.18	14.425	<0.001
**CS+ - GS9**	2.93	0.178	16.47	<0.001
**CS+ - CS-**	2.969	0.178	16.632	<0.001
**CS- - GS2**	-2.777	0.179	-15.533	<0.001
**CS- - GS3**	-2.452	0.179	-13.682	<0.001
**CS- - GS4**	-1.999	0.177	-11.268	<0.001
**CS- - GS5**	-1.619	0.179	-9.018	<0.001
**CS- - GS6**	-1.331	0.179	-7.484	<0.001
**CS- - GS7**	-0.635	0.178	-3.564	0.005

Statistics Smoker manipulation groups without non-smoking individuals

In order to test the robustness of the statistical results, we examined the effect of smoking manipulations between smoking individuals, only.

Fear Acquisition

US expectancy results were analysed with the mixed-model in R.

We found a main effect of stimulus (F(1,2979.87)=42.43,p<0.001) with higher US expectancy for the CS+ as compared to the CS- (CS+-CS-:estimate=3.76,SE=0.097,z-ratio=38.790,p_{corr}<0.001). Furthermore, we found a main effect of block (F(2,2981.64)=13.88,p<0.001) with an increasing US expectancy from block 1 to block 2 and block3 (block1–block2:estimate=-0.5612,SE=0.119,z-ratio=-4.726,p_{corr}<0.001; block1–block3: estimate=-0.6474,SE=0.119,z-ratio=-5.44,p_{corr}<0.001) and a stimulus by block interaction (F(2,2977.7)=40.878,p<0.001). The interaction consisted of higher differentiation between the

CS+ and the CS- in block 2 when compared to block 1 (t(1344)=12.06,p_{corr}<0.001) as well as in block 3 when compared to block 2(t(1344)=3.11,p_{corr}=0.002). Additionally, the analyses revealed a block by group interaction (block*smoker groups: F(4,2977.63)=3.06,p<0.0158), but follow-up post-hoc tests revealed no group differences.
Fear Ratings were analysed in jasp. We found a stimulus main effect (F(1,134)=41.04,p<0.001) with higher Fear Ratings for the CS+ as compared to the CS- (t(548)=6.725,p_{corr}<0.001). Furthermore, we found a main effect of time (F(1,134)=53.486,p<0.001) with an increase in Fear Ratings from pre ACQ to post ACQ (pre–post:t(548)=5.79,p_{corr}<0.001) and a stimulus by time interaction (F(1,134)=69.198,p<0.001). The interaction consisted of higher differentiation between the CS+ and the CS- post ACQ when compared to pre ACQ (t(274)=7.144,p_{corr}<0.001).

Generalization test
We found a stimulus main effect (F(9,2431.4)=16.759,p<0.001) with higher US expectancy for the CS+ when compared to the generalization stimuli from GS3 to CS- (minimal t-value: t(2432)=2.969,p_{corr}=0.042) and lower US expectancy for the CS- as compared to CS+ to GS6 (maximal t-value: t(2432)=-4.032,p_{corr}=0.002).

Table S3: Day 2 US expectancy post-hoc tests between stimuli for smoker manipulation groups without non-smoking individuals. Results were corrected for multiple comparisons using Bonferroni-Holms method.

stimuli	t	df	p_{corr}
**CS+ - GS3**	2.969	2432	0.042
**CS+ - GS4**	3.649	2432	0.005
**CS+ - GS5**	6.667	2432	<0.001
**CS+ - GS6**	8.982	2432	<0.001
**CS+ - GS7**	11.371	2432	<0.001
**CS+ - GS8**	11.651	2432	<0.001
**CS+ - GS9**	13.39	2431	<0.001
**CS+ - CS-**	13.083	2432	<0.001
**CS- - GS2**	-12.288	2432	<0.001
**CS- - GS3**	-10.021	2432	<0.001
**CS- - GS4**	-9.43	2431	<0.001
**CS- - GS5**	-6.233	2432	<0.001
**CS- - GS6**	-4.032	2432	0.002

We found a stimulus main effect (F(1,135)=46.204,p<0.001) with higher Fear Ratings for the CS+ as compared to the CS- (t(550)=8.266,p_{corr}<0.001). Furthermore, we found a main effect of time (F(1,135)=4.983,p=0.016) with a trend towards a decrease in Fear Ratings from pre EXT to post EXT (pre–post:t(505)=1.846,p_{corr}=0.065). Additionally, we found an interaction of stimulus by time (F(1,153)=7.237,p=0.008). The interaction consisted of higher differentiation between the CS+ and the CS- pre ACQ when compared to post ACQ (t(274)=2.137,p_{corr}=0.034).

Statistics Fagerström
We checked both US expectancy and fear ratings for an effect of nicotine dependence with the Fagerström test for nicotine dependence (FTND). The score of the FTND was included into the model (lmer (RatingResults~(1|participants) +stimulus*time*group+fagerström)). The group factor that is included in the model includes the three groups with smokers. We found no main effect of the Fagerström score for US expectancy rating on day 1 (F(9,125.12)=0.409,p=0.928) or day 2 (F(1,133.91)=1.213,p=0.273). Also we found no main effect of the Fagerström score for fear rating on day 1 (F(1,133.38)=0.958,p=0.3295) or day 2

(F(1,134)=1.837,p=0.178). When checking for interactions of the FTND with our fixed effects in the model, we found a trend towards a stimulus by group by FTND interaction (F(2,2964.54)=2.532,p=0.08). For further analysis, we calculated correlation coefficients between the differential US expectancy rating on day 1 and the FTND for each group separately. As the data shows no normal distribution, we used spearman's rho for correlation analysis. Only group 4 showed a correlation between US expectancy rating and FTND (rho=0.163,p<0.011). No correlation between US expectancy on day 1 and the FTND was found for group 2 and group 3 (group 2:rho=-0.049,p=0.282; group 3:rho=-0.016,p=0.722).

Statistic Withdrawal

We checked both US expectancy and fear ratings for an effect of withdrawal. The score of withdrawal symptoms was included into the model (lmer (RatingResults~(1|participants) +stimuli*time*group+withdrawal)). The group factor that is included in the model includes the participants of group 2 and the regrouped participants into group 3. Statistics has only been calculated for the Generalization test, as participants were only asked to take a smoking break between day 1 and day 2 and there should be no effect of this smoking manipulation on day 1. We found no main effect of the withdrawal for US expectancy rating (F(1,66.99)=0.945,p=0.334) or fear rating (F(1,67)=0.121,p=0.729) on day 2.

Statistic sex

We checked both US expectancy and fear ratings for an effect of sex of participants. Sex was included into the model (lmer (RatingResults~(1|participants) +stimuli*time*group*sex)). The group factor that is included in the model is smoker vs. non-smoker.

We found a main effect of sex for US expectancy rating for day 1 (F(1,1139)=6.389, p=0.012), but follow up post-hoc tests did not reveal any differences. Additionally, we found a stimulus by sex interaction (F(1,4376.1)=5.895, p = 0.015), which consisted of an increased US expectancy of females towards the CS- (CS-/male - CS-/female: estimate=-0.416, SE=0.180, z-ratio=-2.306, p_{corr}=0.042). Also we found a trend towards a group by sex interaction (F(1,1145.3)=2.733, p = 0.099). The follow up post-hoc test revealed an increased US expectancy of smoking females, when compared to non-smoking males (non-smoker/male – smoker/female: estimate=-0.482, SE=0.243, z-ratio=-1.986, p_{corr}=0.047). We found no further main effect or interaction of sex on the US expectancy on day 2, or the fear rating on both days.

Statistics reaction time

We checked US expectancy ratings on both days in regard of the participant's reaction time. We included reaction time as dependent variable into our model. Stimulus, block and group (smoker vs. non-smoker) were included as fixed effects into the model (lmer (reactiontime~ stimulus*block*group)). On day 1 we found a trend towards a main effect of stimulus (F(1,4383.5)=3.10,p=0.078), but follow up post-hoc tests showed no difference of reaction time between CS+ and CS-. Additionally we found a main effect of block (F(2,4384.90)=11.667,p<0.001). Follow up post-hoc tests revealed that subjects were rating faster over time (block 1–block 2: estimate=0.316,SE=0.0346,z-ratio=9.118,p_{corr}<0.001; block 2 – block 3:estimate=0.063,SE=0.0346,z-ratio=1.805,p_{corr}=0.071). On day 2 we found no differences regarding the reaction time.

Statistic STAI-T

We checked trait anxiety with the State-Trait Anxiety Inventory (STAI-T). An ANOVA showed no effect of group for the smoker manipulation groups (F(3,198)=0.805,p=0.492). An independent sample t-test revealed also no differences for smokers vs. non-smokers (t(200)=1.541,p=0.125).

Statistics age

We checked both US expectancy and fear ratings for an effect of age of the participants. We added age to the model (lmer (RatingResults~(1|participants) +stimuli*time*group*age) for the analysis of US expectancy and we added age as between subject factor to the type 3 rmANOVA in jasp for the analysis of the fear ratings. The group factor that is included into the model is smoker vs. non-smoker. We found no main effect or interaction of age on either day for US expectancy and fear rating. Inclusion of this variable as separate covariates still yielded robust results of our group differences.

Statistics alcohol and coffee consumption

We checked both US expectancy and fear ratings for an effect of alcohol or coffee consumption of the participants. Subjects stated their alcohol consumption as number of glasses per week and coffee consumption as number of cups per day. Two subjects were excluded from the coffee consumption analysis, because of unrealistic declarations of their coffee consumption (14 cups/day). We added either alcohol or coffee to the model (lmer (RatingResults~(1|participants) +stimuli*time*group*consumption) for the analysis of US expectancy and we added alcohol or coffee consumption as between subject factors to the type 3 rmANOVA in jasp for the analysis of the fear ratings. The group factor that is included into the model is smoker vs. non-smoker. We found no main effect of either alcohol or coffee consumption on either day for US expectancy and fear rating. Nevertheless, we found for the US expectancy ratings on day 1 a stimulus by block by group by coffee consumption interaction ($F(2,4347.4)=4.042$, $p=0.0176$). The interaction reflects increasing differentiation between the CSs with increasing amount of coffee in smokers ($r=0.1$, $p=0.028$), whereas this relationship is not existing (or even the opposite) in non-smoking individuals ($r=-0.076$, $p=0.251$). This difference between groups is most pronounced within the first block during acquisition training ($z = 2.212$, $p = 0.027$). Importantly, we found no effect of coffee consumption on the retrieval or generalisation of CS-responses on day 2, where we observed the most pronounced difference between smoking and non-smoking participants.

These control analyses that included alcohol consumption and coffee consumption as variables consistently revealed smoking status as the significant predictor across measurements.

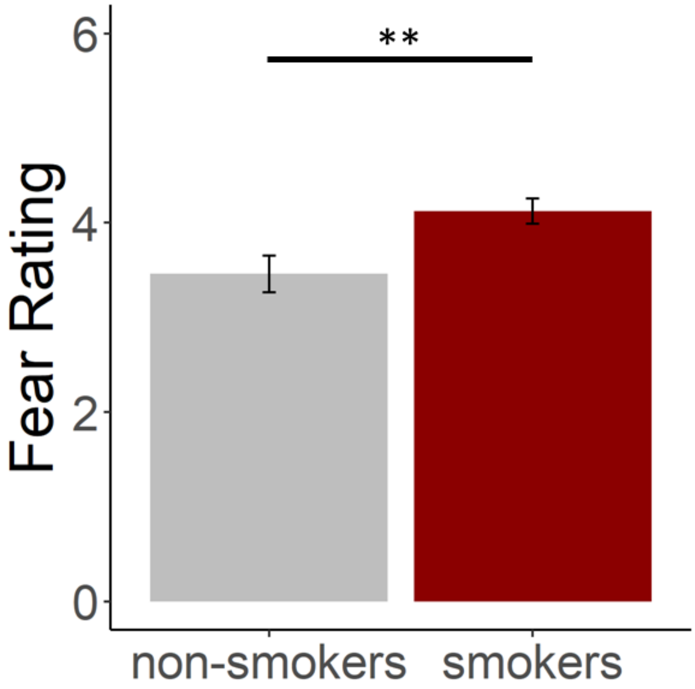

Figure S1: Main effect of group (smokers vs. non-smokers) regarding the fear rating on day1. We found an increased fear rating in smokers, when compared to non-smokers. [**] indicates p<0.01.

Supplementary Material & Methods

Table S4: Day 2 US expectancy post-hoc tests between stimuli in the non-smoking group. Based on these tests, CS like stimuli groups were defined: “CS+ like” (i.e., different from the CS- and not from the CS+ (CS+, GS2, GS3)) and “CS- like” (i.e., different from the CS+ and not from the CS- (GS8, GS9, CS-)). Results were corrected for multiple comparisons using Bonferroni-Holms method.

	stimuli	Estimate	SE	df	t-ratio	p_{corr}
**CS+ like**	CS+ - GS2	0.219	0.276	1144	0.795	1
	CS+ - GS3	0.412	0.276	1144	1.495	1
	CS+ - GS4	1.179	0.273	1144	4.322	<.001
	CS+ - GS5	1.289	0.276	1145	4.674	<.001
	CS+ - GS6	1.399	0.273	1144	5.121	<.001
	CS+ - GS7	2.299	0.274	1144	8.377	<.001
	CS+ - GS8	2.753	0.277	1145	9.944	<.001
	CS+ - GS9	3.068	0.273	1144	11.250	<.001
	CS+ - CS-	3.223	0.275	1145	11.740	<.001
	CS- - GS2	-3.004	0.275	1145	-10.918	<.001
	CS- - GS3	-2.811	0.275	1145	-10.213	<.001
	CS- - GS4	-2.044	0.272	1144	-7.510	<.001
	CS- - GS5	-1.935	0.275	1145	-7.031	<.001
	CS- - GS6	-1.824	0.273	1144	-6.688	<.001
	CS- - GS7	-0.925	0.274	1145	-3.375	0.012
**CS- like**	CS- - GS8	-0.471	0.276	1145	-1.703	0.889
	CS- - GS9	-0.155	0.272	1144	-0.570	0.569

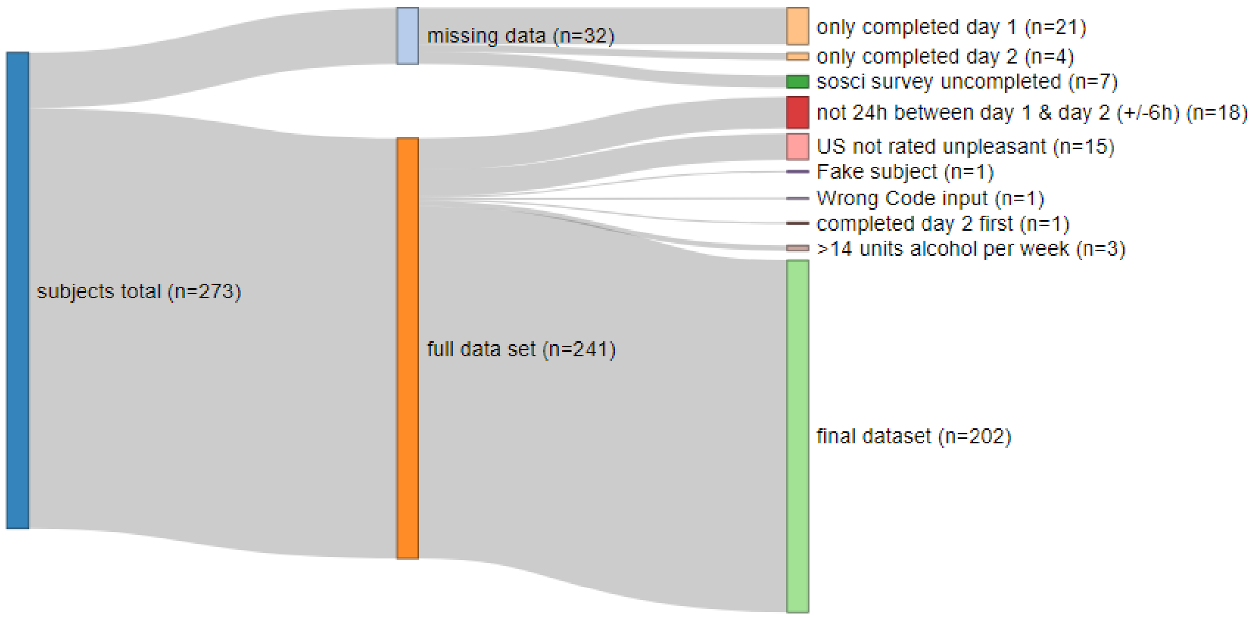

Figure S2: Participant exclusion. From the total of 273 subjects that had been recruited, 241 generated a full data set and finally 202 could be included into the final data set. Main reasons for exclusion by numbers of participants were missing data, participation outside of set time frame and participants that rated the US as not unpleasant.

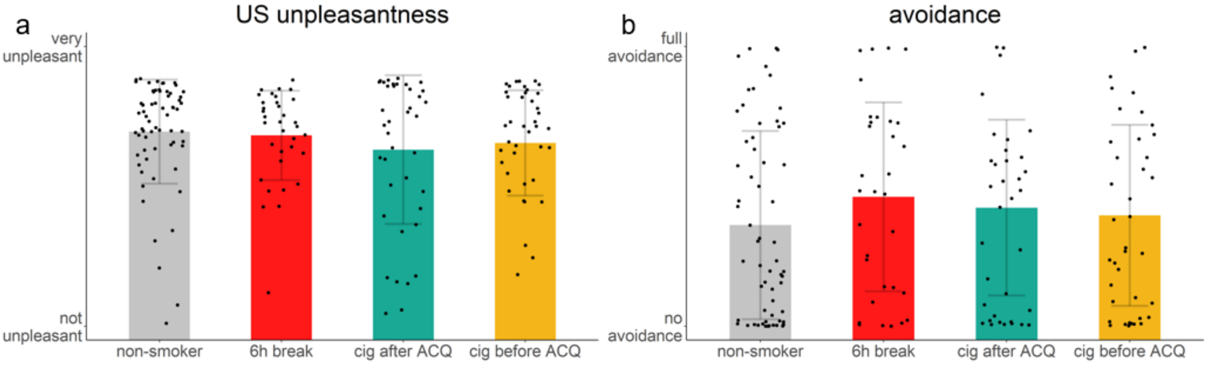

Figure S3: a) US unpleasantness per group. Individual representations in this figure indicate the difference between the mean of the three US and nUS ratings. Subjects with a difference ≤ 1 are already excluded. No difference between the experimental groups were found regarding their rating of US unpleasantness (F(3,173)=1.034, p = 0.379). b) US avoidance per group. Subjects were asked if they avoided to look at the screen when the US was presented on a scale from full avoidance to no avoidance. No difference between the experimental groups were found regarding their rating of US avoidance (F(3,165)=0.693, p=0.558).
